# Some Observations on the Pathology and Prevailing Diseases of Hot Climates

**Published:** 1805-06-01

**Authors:** A. Pearson

**Affiliations:** Member of the London College of Surgeons, and Surgeon in the Service of the East India Company


### Medical and Phyfical Journal.

VOL. XIII.] JUNE 1, 1805. [NO. LXXVI.

*Printed for R. PHILLIPS, by W. Thorne, Red Lion Court, Fleet Street, London.*

### SOME OBSERVATIONS ON THE PATHOLOGY AND PREVAILING DISEASES OF HOT CLIMATES. By A. PEARSON, *Member of the London College of Surgeons, and Surgeon in the Service of the East India Company.*

( Continued from vol. xi. pp. 200—267. )

IT may not appear to come within the description of the diseases of hot climates, to consider scurvy; but what will be offered upon that subject, relates to a modification of the disease, which as far as I can learn is peculiar to such climates, or affects those who have resided in them previously.

The attention of the writer was drawn to this subject by the disease in question having occurred in an East India ship in which he was employed as surgeon; he knows that it has appeared in many others during the late war; and as, both in its appearance and progress, it differs from the disease as usually seen, he expected before this time to have met with an account of it; a detail of the leading symptoms will point out in what respects it resembled or differed from the disease as generally described. It occurred during a homeward passage from China to England; there were from first to last about one hundred and forty affected with it; eight died of the disease; and on the ship's arrival at St. Helena, there was a necessity for landing eighty; these were recovered by the refreshments there; and by the use of lime-juice, the disease was kept stationary till the ship's arrival in England.

### GENERAL SYMPTOMS.

First stage.—Slight febrile affection indicated by the skin and pulse, facies scorbutica, morning lassitude, general pains, breathlessness on motion, and swelling of the extremities.

Second stage.—Increase of the swellings both in the trunk and lower extremities, greater in the former in the morning, and in the latter at night. Dyspnœa ascribed to a pain in the breast felt most severely under the sternum, or in the left side. This was occasionally aggravated and most severely felt when the swelling receded, or during the night; on such occasions a sense of tightness across the breast, sometimes of a lump moving its place, excited such a dyspnœa as to render the patient apprehensive of suffocation. Stiffness of the flexors, pain along the muscles, obstinate costiveness, vomiting of cathartics, and voiding of enemata without effeot.

Third stage.—The swellings receded from the extremities, at first occasionally, and afterwards permanently, when that in the abdomen became greater, and the difficulty of breathing was increased. Ineffectual tenesmus, much debility, cardialgia, severe retchings, pain of the breast of a more acute kind than before, a sense of heavy griping in the abdomen, which seemed to occasion great agony, with constant restlessness and inability to sleep; these last were considered as immediate forerunners of a fatal event.

### Symptoms not general.

The itching, swelling and bleeding of the gums, their putrescence, and the spots and blotches of the skin, which have been reckoned pathognomic of the disease, were totally wanting in the worst cases, and in more than two-thirds of the remainder. In the greater part even of these instances where they occurred, they could only be detected by hard rubbing of the gums, or a close inspection of the skin. In three cases only did the disease advance in the common form, rapidly indeed, but with a comparatively advantageous progress. There were no cases of scorbutic ulcer; and in two persons who had ulcers of the leg at the time of the attack, although they assumed somewhat of the scorbutic appearance, there was no shooting of fungus. Anorexia and a quickened pulse were pretty general, and worms of the lumbrici kind, and symptoms of them, so much as to form a remarkable addition to the other complaints. In three or four places febrile complaints combined with and run into the disease; and in one case a paroxysm of fever attended the attack of the fatal symptoms: in this person the skin had been of an icteritious colour for some time before. Hæmorrhages from the nose occurred in three instances; from the lungs in two; and from the stomach in one. Several had a sense of contraction in the right side of the œsophagus, whether symptomatic of the worms or not I cannot determine. One person felt a singular instability and vacillation of limbs; in in another there was a permanent dilatation of the pupils, with impaired vision. In one a bilious diarrhœa coming on spontaneously, relieved the symptoms much. In another the affection of the breast went off, and an ischuria took place, so complete as to render the use of the catheter necessary, and these affections alternated repeatedly.

The period in which the disease run its course differed much in various individuals. From a week to a month, dating from the time when they ceased to do duty. On dissection of a person who died of the disease, the principal, and indeed only morbid appearances, were a great quantity of serum in the cellular membrane, some also in the cavity of the abdomen; lumbrici ascarides unusually large and numerous in the ilium. The liver was considerably enlarged in its substance, but with very little organic disease; in one or two places a slight appearance of suppuration, resembling in consistence and size the matter of a very small furunculus. Swellings of the scrotum were not unfrequent, but were more common, and increased for a time in size while the patients were recovering, which they did very rapidly after arriving at St. Helena, under a fresh and vegetable diet with a plentiful supply of limes. The weather had suddenly altered from being warm in the S. E. trade wind, to cold, when doubling the Cape of Good Hope.

It will be at once seen that the symptoms differ materially from those which are generally detailed, not corresponding to the definition of Dr. Cullen,* or to the corrected and more generally applicable one of Dr. Trotter,† but it is impossible to question the identity of the disease. It may be of use to those in situations exposed to it to be aware of the possibility of its approach in such a form, and also that it is a more formidable species of the disease, than that which is generally met with. That which approaches nearest to it is the account of a scurvy making its appearance in the American ship of war in the East Indies, given at p. 278 of the third edition of Dr. Lind's Treatise. From having had but a small supply of lime-juice at first,

* In regione frigida post victum putrescentem salitum, ex animalibus confectum, deficiente simul materia vegetabili recente; asthenia stomacace; in cute maculæ diversicolores, plerumque livescentes præsertim ad pilorum radices.

† Asthenia, stomacace, in cute maculæ diversicolores, plerumque livescentes; deficiente simul vegetabili materie recente, eundemque vehemente ingerendi desiderio.

from the length of the preceding voyage, and from having left China at a season when it was not procurable, we were without a sufficiency of that essential remedy. The best substitute we could think of was tamarinds, of which forty pounds had been provided, and the infusion by itself, or in combination with decoction of sassafras, or wine and sugar, given with benefit and effect. Purgatives and the body kept lax by tart. solub. p. rhæi p. inf. sennæ, were necessary. Sudorifics sometimes afforded a temporay relief. Nitre or camphire. Aq. ammonia with a few drops of tinct. opii given in warm wine and water till a perspiration broke out, and keeping it up by diluents, were of use in relieving the affection of the breast, whenever it became much aggravated. Blisters and squill medicines also seemed useful. Vermifuge medicines, where indicated, P. stanni; sem. santon. & calomel. Fomentations, ol. palmæ, and s. v. camphorat. for the stiffened flexors. The disease was kept under during the passage from St. Helena to England by lime-juice only, given from ℥ij to ℥vj. daily; no doubt the concrete acid might be an useful substitute, but the nearer the acid is to the state in which it is while in the fruit, I apprehend it will prove the more effectual. A supply of fresh provision from the commander's table was one of our most efficacious resources.*

From

---

* In order to convey an idea of the state of constitution of those affected with this disease, the following statement is offered, premising that a merchant ship's company obtained in war time must necessarily have been subjected to many depressing influences.

The ship in question sailed from Spithead, April 6, 1795. Ship's company 175 men, inclusive number on board 210. Were eight or nine weeks on the passage to St. Helena; remained there till July, receiving supplies of fresh provisions and vegetables, but not regularly.

Left St. Helena with four hundred troops embarked; a healthy passage of four weeks to the Cape of Good Hope, where, from its being then in a state of siege, no refreshments were procurable.

Proceeded to Madras with only the ship's company on board; arrived in the end of September.

Remained at Madras a fortnight, receiving the customary refreshments there, which are plentiful, but not of the best description.

Left Madras in the middle of October, attached to the expedition under Admiral Rainier for the reduction of the Dutch settlements, having on board upwards of three hundred native troops.

From this time to the end of December, passed a fortnight at Penang Malacca, when the ship sailed from the last mentioned harbour, and was nearly three months at sea, by an eastern passage to her ultimate destination in China, having only the ship's company and a few invalids on board.

From the beginning of April to the middle of May which is an unhealthy season of the year, the ship was anchored at Second Bar in the river of

Canton,

From the statement contained in the note may be inferred what causes predisposed and were favorable to the attack of the disease. I am not without suspicion, that the mercurial treatment of these complaints, which prevailed previously to the ship's departure from China, may have deserved to be enumerated with them. That there is reason to apprehend such an effect from it, will not be an objection to the having recourse to it in cases of urgency, while it may be allowed in connexion with other practical considerations, to limit its administration in those which are not so, or to afford a caution with regard to the indiscriminate use of calomel as a cathartic, when other remedies of that class will equally well answer the purpose.

It seems unnecessary to proceed farther in detailing the treatment, as the curative means pointed out by Lind and Trotter are equally applicable to this as to the forms of the disease which they describe; and it would be well for our profession, if we could always have the aid of as faithful observers, and judicious guides of practice.

There would be still less chance of offering any thing satisfactory as to the proximate cause or ratio symptomatum. The operation of the remote causes may be supposed to induce such a state of the vital powers and actions as may be accompanied by a debility and relaxation of fibre; by an altered state of the circulating fluid, and an imperfect oxygenation of it; or a defective action of the lymphatic and venous absorbents; but no one of these can be considered but as effects of the general state, and not as a proximate cause to account for the symptoms, or why so great and general a failure of the functions so quickly takes place, from a state of general health, and without pyrexia.

I would likewise refer to these authors, and to a consideration of the remote causes, for what are likely to be the best means of prevention. Dry air by means of stoves, warm clothing, abundance of water, bread instead of rice, and yams or potatoes, served to the ship's company with their salt provisions; fresh provisions, vegetables and fruits where attainable. These resources are as well known as their beneficial effects are limitted; and that the havock

 formerly

Canton, a very insalubrious situation, during which time fever and dysentery were universal among the ship's company.

With their constitutions injured by these causes and preceding debauchery, their provisions also the worse for having been long kept, there was another eastern passage to be undertaken; and in the course of it, upwards of five months to St. Helena, the disease in question broke out.

formerly made by the scurvy among our fleets is now diminished, proceeds not so much from our knowing better how to treat the disease, as that an improved state of discipline and attention to the health of the men have rendered its occurrence more rare than in former times.

The conviction of this fact, and other considerations, lead to the expression of a wish that the seamen in the merchant service might become objects of the care and attention of the legislature, or of such members of the community as can best obtain its interference in their behalf. There is actually no consistent code of laws, or set of regulations to secure their well being and proper treatment as men, and at the same time to act as a due restraint upon the evil propensities and injurious habits which are too prevalent among them, owing perhaps to the want of some such protecting influence.

In the preceding series of observations, I have adverted to the diseases which are most generally met with in hot climates. Tetanus, supposed to be frequent, has not often come under my observation. In one case, where it succeeded an operation, the patient died; in two others they recovered from the immediate effects of the disease. I am at a loss to what to ascribe any beneficial effect in these cases, as every means whose operation did not interfere with each other were made trial of. Purging with calomel in the beginning; plasters of opium and camphor to the seat of the pain in the sternum; friction with liniments of the stimulant kind on the jaws and neck; above all, frequent use of the warm bath from 92° to 96° of Fahrenheit, for fifteen or twenty minutes at a time, and friction on the affected muscles with mercurial ointment till ptyalism is brought on. Musk, castor and opium internally, especially before the coming on of the convulsive or spastic affections of the muscles.

The dry belly-ache is said to be a frequent disease in hot climates, so much so at particular times, as to appear to be epidemic; and certainly, where I have met with it, several have been affected at the same time, but that probably arose from an identity in circumstances of situation and habits. When a train of symptoms resembling it occurs, it will be useful to ascertain whether it is the colica stercorea or that arising from lead; and the first indication to be attended to, is to evacuate the great intestines by terebinthinate, purging or emollient glysters; their use and operation are indispensable, and will be much aided by the warm bath, while, at the same time, the other means pointed out by authors may be practised.

It

It has beeome a question I believe whether coup de soleil ought to be treated by evacuation, or as a disease of debility. I have not data from which to decide, nor to form an opinion for myself better than a probable one. In the few instances which have occurred, the symptoms of congestion in the head and increased action were so obvious, as to seem to the most inexperienced observer to require depletion and the antiphlogistic treatment. At the same time I am convinced, that men from fatigue and exposure to the rays of the sun in a hot climate, often fall down, as it is said, from the effects of a coup de soleil, when it is in fact a syncope. It cannot be difficult to discriminate betwixt these two cases, nor to determine upon which is the injurious, and which the proper mode of treatment.

Mr. F. aged about nineteen, had been intoxicated the preceding evening, and slept exposed to the rays of the morning sun with his head uncovered. Thermometer at 82°. He was with difficulty awakened, when he was attacked with strong delirium and occasional convulsions. Skin very hot; face flushed; pulse full and quick; eyes blood-shot. Blood was taken to nearly fourteen ounces, which, as it flowed from the vein, communicated nearly a scalding sensation to the fingers; the head, face, and neck were bathed with a solution of muriat of ammonia in vinegar; he was ordered inf. sennæ and tamarind, afterwards a purge with calomel; antiphlogistic treatment, and infusion of tamarinds for drink. During two or three days succeeding the attack he had occasional relapses of the delirium and convulsions, afterwards he was free from complaint, but much weakened. Such a train of symptoms as the above I would expect to meet with in a person who had suffered a coup de soleil; and there can be little question, that if the patient had remained a short while longer unnoticed, that he would have awoke no more.

As an addition to the collection of facts towards a history of the late epidemical catarrh, which have been collected by Dr. Beddoes, and published in the Medical and Physical Journal, I beg leave to offer the following sketch of the disease, as it appeared in China; whence there is every reason for supposing the influenza to have originated, and where, from being peculiarly situated, I had during the month of September, 1800, a great many cases under treatment.

1800. At Whampsa, in China.

About the 25th of September, several of the people were attacked with a catarrhal fever, which quickly spread thro' the ship's company. As it was so general, and at the same time so uniform in its symptoms, instead of detailing every particular case, I will attempt a description of it, as it appeared, and was suffered.

Having so many to attend to while I was myself pretty severely affected, left but little leisure to arrange or commit to paper observations in any other form; a diary was kept of the names and prescriptions. I shall first mention the most general appearances of them, and then advert to any particulars which a difference of constitution or other circumstances might have produced.

Three days were the limits of its continuance in the febrile form, but the pectoral symptoms were of longer duration.

Its attack was sudden, with an uncommon degree of lassitude and sense of weakness, with transient chills. These were followed by a coryza, severe cough, pain of the breast, dyspnœa, sense of stuffing, and scanty expectoration. The throat was affected in about two-thirds of those attacked. There was head-ach, principally of the temples, with pain of the eye-balls, nausea, anorexia, and thirst at all times; the tongue was covered with a white crust, and the mouth felt clammy. There was a febrile exacerbation occurring twice in the twenty-four hours, at its height about noon and midnight; the former severest: in some these were preceded by chills. In general, the increased heat, sense of weakness, and frequency of pulse, came on without any such warning. In my own case the fever was always preceded by slight rigors; the pulse was always quick; in the febrile state often upwards of 120, regular but not strong in those of the most robust habits, in others weak and soft: at that time too there was frequently a tendency to a hurry of thought and incoherence, with flushing of the face, and throbbing of the temporal arteries. In more than one case bleeding at the nose occurred, which relieved those symptoms, and the fever abated.

At the beginning of the disease the belly was costive; after it run its course, it was easily moved by laxatives, or a spontaneous diarrhœa came on; in either case the diarrhœas were bilious and acrid, and produced a burning heat about the anus. At the same period too the expectoration became copious, purulent looking, and often tinged with blood, the cough was even more frequent and troublesome; and tho' the appetite returned and the strength was was increasing, these symptoms continued for a week or a fortnight, after the abatement of the febrile symptoms; swelling and stiffness of the inguinal and axillary glands were frequent as the fever abated, and sometimes suppurated; in several there was otalgia, and in a few instances suppuration of the internal ear.

There were only six of the ship's company exempted from the attack of this disease, who had been either recently ill, or taking mercury.

The treatment was nearly as uniform as the symptoms of the disease. An emetic as soon as possible after the attack of the disease, purgatives of calomel and Glauber salts, afterwards frequent gentle laxatives, nitrous mixture with antimonial wine, or a mixture with spermaceti and acetum scilliticum, as the febrile or pectoral symptoms were predominant. Opiates during the febrile stage were uniformly hurtful, increasing the heat, irritation, and tendency to delirium, without procuring sleep; afterwards, when the primæ viæ were cleared, they were used with advantage in the evening doses of the spermaceti mixture.

Blood-letting was practised in only one case, where it seemed to be indicated by the severity of the pulmonary affection; but the increased sense of weakness, and other unfavourable symptoms which followed its use, prevented any farther trial of it. The prostration of strength, the weakness and quickness of pulse, seemed to indicate the use of bark, and it was given in some cases where the remission was most complete and the symptoms of pulmonary congestion least urgent, but with the effect of increasing every symptom of febrile irritation as well as the pain of the breast and difficulty of expectoration. It was not tried again till the state of convalescence rendered its administration adviseable and useful; calomel, so frequently had recourse to in febrile affections, appeared to have little advantage over other laxatives. In my own case, and in several others, after the fever was gone off, where symptoms of visceral obstruction had before existed, or were brought on, I gave one grain of calomel for ten or twelve successive nights, and two ounces of a solution of ant. tart. gr. ij. in aq. ℔jß. three times a day; this seemed to be useful in restoring the secretions of the bowels, and purgatives given afterwards brought off much bile and vitiated mucus; the convalescence was in general favorable. The same disease prevailed among the Lascars of some country ships, where its progress was similar, tho' the symptoms were milder. There had been an epidemical catarrh among the Chinese, which had nearly ceased before fore our arrival; and among them, as well as the Portuguese at Macao, had been frequently fatal.

During some time before there had been wet weather, afterwards a few days when it was cool and the thermometer at 72°. When the disease came on, the winds were southerly; the thermometer 83° ad 90°. By the 15th of October the ship was again tolerably healthy.

There appear to me some circumstances in the following dissection, worth relating, though I do not see how it leads to any inferences of practical utility.

Mr. I. M. aged about twenty-two, was found dead in his bed at eight o'clock in the morning; it is probable he expired but a short time before, as the body had not entirely lost its temperature. He had on the preceding evening made some slight complaint of nausea, and pain in the epigastrium, but not in urgency so as to be particularly noticed. He was heard, by a gentleman who slept near him, to moan and be disturbed during the night; as that was frequently, and indeed generally the case, no inquiry was made; the usual means of recovery were had recourse to with no effect. It was judged proper to investigate the cause of the death by opening the body, which was done in the presence of two other surgeons. We were struck at first with the size of the head, and especially of the parietal bones being so disproportionately large. The membranes of the brain adhered more strongly than is usual to the cranium, and a considerable quantity of serum exuded on sawing it through. In all the course of the longitudinal sinus, the membranes were of a denser consistence than is common, hardening more and more till they arrived at the vertex, where, in the course of the sinus, and enclosed between the membranes, there was found a complete ossification of the size of a thumb-nail, a ragged point of which had pierced the internal membrane, and as it lay must have entered into and vellicated the brain. The membrane was in a similar state backwards to the transverse septum; and at the going off of the lateral sinuses, there was an ossification of the same size as the other, no part of which had penetrated the membranes. The brain appeared very large, and the membranes at this place adhered more strongly to it than is usual. In attempting to separate them, the instrument pierced one of the ventricles, and an unusual quantity of clear serum escaped; in that and the other ventricle, not less than ten ounces had been contained; the brain then collapsed. Nothing particular was to be felt in the plexus choroides, nor

2 any any discernible alteration in the thalami of the optic nerves, neither indeed was there in the substance or consistence of the brain, except that the cineritious part appeared to be in a greater than common proportion.

It must appear surprising, that so much disease should exist without a greater derangement in the functions of the sensorium. Neither the external nor internal senses had appeared to be much impaired. He had been educated for the medical profession liberally, and though his acquirements were not in proportion, they were not remarkably deficient. He was subject to head-achs, and occasional lowness of spirits. On any unwonted excess he seemed at the time and for a few days afterwards to have a tendency to mania. His gesture and gait appeared to be vacillatory and unsteady; he had a correct musical ear, and played on the German flute with uncommon taste and execution. The eyes were lighter coloured than I ever saw them in any other person, and the pupils were always much dilated. From the whole of his skin there proceeded a peculiar and disagreeable fetor.

It is almost unnecessary to state that the thoughts submitted to the public through the medium of the Medical and Physical Journal, in this and three preceding papers, are observations merely, and do by no means aspire to be considered as treatises on the several subjects; that they do not claim to be founded on peculiar extent of experience, on sagacity of observation, depth of research, or ingenuity of reasoning. If they are found to bear upon points and facts of practical utility, and to furnish any clue to the minds of those members of the profession, who may be situated as the author has been to guide them, in their inquiries or practice, he will deem the obtrusion of these thoughts to be justified, and is willing that the degree in which this aim is accomplished should be held to be the test of their merit or demerit.

